# Explanatory pragmatism: a context-sensitive framework for explainable medical AI

**DOI:** 10.1007/s10676-022-09632-3

**Published:** 2022-02-28

**Authors:** Rune Nyrup, Diana Robinson

**Affiliations:** 1 Leverhulme Centre for the Future of Intelligence, University of Cambridge, Cambridge, UK; 2 Department of History and Philosophy of Science, University of Cambridge, Cambridge, UK; 3 Department of Computer Science, University of Cambridge, Cambridge, UK; 4 Microsoft Research, Cambridge, UK

**Keywords:** Explainable artificial intelligence, XAI, Medical artificial intelligence, Explanation, Understanding, Ethics of artificial intelligence

## Abstract

Explainable artificial intelligence (XAI) is an emerging, multidisciplinary field of research that seeks to develop methods and tools for making AI systems more explainable or interpretable. XAI researchers increasingly recognise explainability as a context-, audience- and purpose-sensitive phenomenon, rather than a single well-defined property that can be directly measured and optimised. However, since there is currently no overarching definition of explainability, this poses a risk of miscommunication between the many different researchers within this multidisciplinary space. This is the problem we seek to address in this paper. We outline a framework, called *Explanatory Pragmatism*, which we argue has two attractive features. First, it allows us to conceptualise explainability in explicitly context-, audience- and purpose-relative terms, while retaining a unified underlying definition of explainability. Second, it makes visible any normative disagreements that may underpin conflicting claims about explainability regarding the purposes for which explanations are sought. Third, it allows us to distinguish several dimensions of AI explainability. We illustrate this framework by applying it to a case study involving a machine learning model for predicting whether patients suffering disorders of consciousness were likely to recover consciousness.

## Introduction

Medicine and healthcare are often highlighted as some of the most promising domains of application for artificial intelligence (AI). Building on recent breakthroughs in machine learning, medical AI systems are developed to take on increasingly critical roles in assisting with clinical reasoning tasks such as diagnosis, prognosis and treatment decisions.

For example, in one recent study researchers used machine learning to build a prognostic model to predict whether patients at a military hospital in Beijing suffering from disorders of consciousness (DoC) following brain injury would recover within 12 months (Song et al., 2018).^1^ The model takes inputs from fMRI scans and a few clinical details to generate a predicted score on a standard 23-point scale for signs of consciousness, as well as a binary prediction of whether the patient will recover consciousness.^2^ The results were promising: the model achieved 88% accuracy in predicting consciousness recovery on external validation data (including from a different hospital in Guangzhou), with similarly promising true positive and true negative rates. The system was reported by the World Economic Forum as one of the “7 amazing ways artificial intelligence is used in healthcare” (Gray, 2018) and used in the daily operations of the Beijing hospital (Chen, 2018).

We highlight this case for a couple of reasons. First, it illustrates the potential benefits that could be gained from medical applications of machine learning and related technologies. For instance, the model correctly predicted seven out of eight patients in the validation dataset who regained consciousness, despite doctors scoring them below 7 for signs of consciousness, the legal threshold for a family to withdraw life support (Chen, 2018). This also illustrates how machine learning may be relied upon in high-stakes decision-making. When a patient is predicted not to recover, this could lead to life-support being removed or therapeutic interventions being prioritised for other patients (Song et al., 2018, pp. 1-2).

What interests us most about this case, though, is the claim by the authors that their model “also has good interpretability, thereby providing a window to reassure physicians and scientists about the significance of different predictors” (p. 21). This claim is significant, since it addresses one of the major concerns about the increased reliance on AI systems in high-stakes contexts, such as the medical domain, namely that they are “opaque” (Burrell, 2016), “black boxes” (Castelvecchi, 2016), or lacking in “interpretability” or “explainability” (Watson et al., 2019). This concern is taken seriously by many, including policy makers and technology companies. For instance, a recent survey found that 73 out the 84 reviewed AI ethics guidelines proposed ethical principles relating to Transparency (including ‘explainability’, ‘explicability’, ‘understandability’ and ‘interpretability’), making it the most commonly discussed type of principle (Jobin et al., 2019). In response, a growing body of technical AI research has emerged, which seeks to develop methods for making AI systems more “interpretable” or “explainable” (see e.g., Doshi-Velez & Kim, 2017; Biran & Cotton, 2017; Chakraborty et al., 2018; Guidotto et al., 2018; Gunning & Aha, 2019), a field often known as Explainable AI (XAI).^3^

An overarching difficulty in this research is that the end goal of having an explainable or interpretable system lacks a precise definition (Doshi-Velez & Kim, 2017; Lipton, 2017). XAI researchers are increasingly recognising explainability as a context- and audience-sensitive phenomenon, rather than a single mathematically defined property that can be directly measured and optimised (Kim, 2021; Tomsett et al., 2018). The lack of precise definitions is problematic for several reasons. First, for technical researchers it means a constant need for direction verification and course correction to make sure they focus on the right goals and make progress toward them (Kim, 2021). Second, discussions about explainability involve many different groups, including the technical machine learning community, domain experts who are meant to use models, human-computer interaction researchers working to bridge the gap between technical models and users, and regulators and ethicists seeking to establish principled ways to safely oversee these advances. In a multidisciplinary space such as this, having clear definitions of key terms is crucial to ensure productive communication and common goals. In short, the problem is this: how can we conceptualise explainability in a way that is unified enough to allow cross-disciplinary communication, while also capturing its context- and audience-sensitivity?

This is the problem we aim to resolve in this paper. We will outline a framework, called *Explanatory Pragmatism*, which we argue can illuminate some nuances and key questions relevant to evaluating explainability in AI systems, especially in medical applications. We start by briefly reviewing some of the recent developments in the technical research and ethical debates about explainable AI. Next, we propose and defend a general account of what it means for an AI system to be explainable, based on pragmatic accounts of explanation and understanding in philosophy of science. We highlight several attractive features of this framework. First, it allows us to conceptualise explainability in explicitly context-, audience- and purpose-relative terms, while retaining a unified underlying definition of explainability. Second, it makes visible any normative disagreements that may underpin conflicting claims about explainability regarding the purposes for which explanations are sought. Third, it allows us to distinguish several dimensions of AI explainability. Finally, we apply Explanatory Pragmatism to the consciousness recovery case study introduced above, to illustrate its usefulness for distinguishing and analysing different types of explainability in medical AI.

## Current directions in explainable AI

Much progress has been made in the last few years in response to the above concerns about explainability. Early debates tended to focus on black-and-white questions of whether or not AI systems could be explainable and still predictive or whether they should be deployed at all without being fully explainable. For instance, the UK House of Lords report on AI stated that “it is not acceptable to deploy any artificial intelligence system which could have a substantial impact on an individual’s life, unless it can generate a full and satisfactory explanation for the decisions it will take” (House of Lords, 2018, p. 40). Meanwhile, critics, such as Weinberger (2018) and London (2019), rejected blanket requirements for explainability on the grounds that this would risk forgoing the highest possible accuracy, by giving preference to simpler but less accurate models, which in high-stakes medical contexts ultimately would translate into higher morbidity and mortality for patients.

More recently, however, researchers have questioned whether there is in fact an inherent trade-off between explainability and predictive power (Kim, 2021). There has been a proliferation of approaches to improving explainability without necessarily restricting oneself to simpler models. Some current directions in XAI research include verification of explanations (Camburu et al., 2019), making explanations more amenable to human understanding by defining concept-equivalent components rather than explanations based on individual features (e.g. training an algorithm in a pathology context to recognise groups of pixels as glands rather than just pointing to particular regions on the image in isolation) (Cai et al., 2019; Kim et al., 2018), and automatically discovering such concepts (Ghorbani et al., 2019). Some researchers have also started to explore explainability at a system-level, where developers can struggle to explain the behaviour of ensembles of models because of unpredictable or unforeseen interactions between the component models, and suggest new ways of building systems to meet this challenge (Lawrence, 2020; Zittrain, 2019).

More generally, researchers have started to investigate explainability in specific contexts rather than as an abstract desideratum. For instance, in a recent paper in the applied machine learning space, Sendak et al. (2020) built and deployed a tool to detect sepsis. They raise several instructive points in relation to their case study of developing and deploying this model. First, they question whether the purposes for which explainability is being used—e.g. as a way to build trust in machine learning models in medicine or to ensure transparency—have been overemphasised. In their case study, they found that there were other ways of developing trust and accountability, for instance in the ways that teams were designed. These included involving domain stakeholders in the development and acknowledging the labour of interpreting and translating the outputs of the model into clinical practice. They also raised more detailed points about which parts of the model required explainability and which did not. For instance, clinicians were not interested in interpreting the cause of sepsis since the treatment pathways were the same regardless, but they did want to know key facts about the model such as what input data the model used, how it had been validated, and which types of decisions it was designed to support.

Regulatory thinking has also come some distance from the earlier statements, such as the 2018 House of Lords report. For instance, recently released regulations addressing clinical trials for machine learning-based decision support tools include more specific guidelines for researchers and developers aiming to deploy models in the clinical context, including thinking about the skills needed for people to use and understand the models as well as error cases and evaluation contexts (Liu et al., 2020; Heaven, 2020; Genin & Grote, 2021).

Finally, there is an increased focus on the need for conceptual clarification. While ‘explainable AI’ and related terms are widely used in the technical and policy literatures, commentators have highlighted that these terms lack any clear, agreed-upon definition (Besold & Uckelman, 2018; Felten, 2017; Kirsch, 2017; Krishnan, 2019; Lipton, 2017; Selbst & Barocas, 2018; Tomsett et al., 2018; Weller, 2017; Zednik, 2019). As the brief review in this section illustrates, there is considerable variety in what kinds of things are meant to be explained—e.g., models, components of models, individual decisions, interacting ensembles of models, the design process that produced a given system—and the types of features that are claimed to make a given system or model (etc.) explainable. Moreover, when motivating the need for explainability, people highlight many different kinds of problems it is supposed to help alleviate. What people regard as an adequate explanation seems to vary by user, context and purpose.

In response to this apparent disunity, some have doubted the usefulness of the term ‘explainable AI’ (Krishnan, 2019). Others have proposed more contextual accounts of explainability, aiming to accommodate this plurality within a general, unified framework (Besold & Uckelman, 2018; Tomsett et al., 2018; Zednik, 2019).

We subscribe to the latter approach. Being able to compare and contrast different approaches to explainability within a unified framework will be valuable as a means to facilitate communication within and across the different communities working on XAI. Of course, such a framework also needs to be flexible enough to genuinely capture the different aspects of the problem(s). In the next section, we develop an account of explainability which, we argue, achieves just that.

## A framework for explainable AI

A natural starting point for an account of explainability is to ask what counts as a good explanation. We start by outlining an answer to this question, based on some ideas from the philosophical literature on explanation and understanding, before proposing a definition of explainability and comparing it to some extant proposals. We defend our framework against some potential objections in “Potential Objections”.

In recent decades, philosophers of science have increasingly emphasised that scientific explanations vary along several different dimensions, such as the type of information provided (e.g. general laws or local causes), implicit contrast-classes (why did you close *the window* vs. why did you *close* the window), and level of abstraction and idealization (e.g. Jackson & Petit, 1992; Potochnik, 2016; Sterelny, 1996; Weisberg, 2007). Often, for the very same phenomenon, several valid explanations are available. For instance, if we ask why the cheetah is able to reach speeds of up to 120 km/h, the available explanations include: a physiological explanation, highlighting facts about the build, muscle-structure and metabolism of the cheetah’s body; an ecological explanation, highlighting that the cheetah’s ecological niche involves hunting certain kinds of fast prey for which the ability to reach high speed is necessary; and a phylogenetic explanation, which tracks the series of historical speciation events that led to the emergence of the cheetah. Crucially, all three types of explanations are valid and can, depending on purpose and audience, count as the best or most adequate explanation.

Our account of AI explainability draws on two, broadly pragmatist ideas that philosophers of science have proposed to account for this plurality in what counts as good explanations: a communicative view of explanation and an inferentialist view of understanding.

### Communicative view of explanation

It has been noted that the verb ‘explains’ can take at least three different kinds of things as its subject (Craver, 2014, cited by Potochnik, 2016): a fact or entity in the world (“the cold explains his sore throat”), a theory or other representation of the world (“infections are explained by the germ theory”) and an agent (“the doctor explains how the vaccine works”). Existing philosophical theories differ as to which of these uses constitute the most fundamental phenomenon and which are merely derivative. Communicative views prioritise the third use (Franco, 2019; Potochnik, 2016; Wilkenfeld, 2014).

We adopt the following formulation of the communicative view: explanations are communicative acts where an *explainer* conveys some information to an *audience*, in order for that audience to obtain some relevant *understanding*. This contains two ideas. First, that explanations should be conceived as speech acts (Austin, 1962) and thus be evaluated in terms of how well they achieve their communicative function. Second, that the characteristic communicative function of explanations is for the audience to obtain some relevant understanding (Franco, 2019). In other words, the ‘goodness’ of an explanation depends on whether it would, under the right circumstances (the audience is attentive, makes an effort to understand what was said, etc.), help the audience obtain the relevant type of understanding. Explanations can of course be used for other purposes, such as to mislead or manipulate people into trusting the explainer. But these uses are parasitic on the paradigmatic function of explanations, viz. to improve the audience’s understanding, in the same way that lying is parasitic on the fact that assertions are generally assumed to aim at conveying truthful information.

As Wilkenfeld (2014) points out, this view avoids imposing any general constraints on the structure of explanations or the type of information they should cite. Instead, an explanation is functionally defined simply as *the kind of thing that, under the right circumstances, produces the right kind of understanding*. This is how the communicative view builds in the plurality and context-sensitivity noted above, while maintaining an underlying unified notion of explanation (Wilkenfeld, 2014: 3367-69). This is not to say that there are no constraints whatsoever or that all explanations are equally good. Rather, what counts as explanatory (or a good explanation) in a given context depends on what best helps the audience obtain the relevant kind of understanding. The point is that these constraints are derived from what constitutes ‘relevant understanding’, rather than the concept of explanation itself.

### Inferentialist view of understanding

What is relevant understanding, then? As several philosophers have argued, understanding is closely related to the ability to draw relevant practical and theoretical inferences (e.g. de Regt, 2017; Leonelli, 2009; Stuart, 2018; Wilkenfeld, 2013). While understanding (at least of the kind conveyed through explanations) involves having some kind of information or representation of the thing understood, simply knowing a set of facts is insufficient for understanding. A person might know many facts about computers through reading an authoritative textbook: e.g. that the harddisk stores programmes, that most computers require stored programmes to function, that overheating can cause components to break, etc. However, if they are unable to use this information to competently draw inferences like “the computer stopped working because the harddisk broke” or “we should identify potential sources of overheating to prevent this from happening again”, we would be reluctant to say that they *understand* computers.^4^

Notably, ascriptions of understanding are context-sensitive (Kelp, 2015; Wilkenfeld, 2017). When we say that a person “understands” something, we do not simply praise their ability to draw any inferences whatsoever. Rather, the conversational context will implicitly pick out some purpose which in turn determines a class of inferences that are relevant for achieving that purpose. For instance, if Mo says “I understand how my fridge works” in an everyday context, he may simply mean that he knows how to use it to keep food fresh. However, if it breaks and Mo exclaims “I don’t understand this fridge, I wish Jill was here!”, he is making a different purpose salient—namely repairing the fridge—which he thinks Jill is able to achieve. Notice that the context-sensitivity here tracks the conversational context of the speaker, not the subject of understanding-ascriptions. Given the purpose Mo makes salient, whether he is correct to say that Jill understands the fridge depends on whether she is in fact able to repair it, regardless of whether she has any interest or intention of doing so.

### Explanatory pragmatism

Putting together the above ideas, we obtain the following account of good explanations: a communicative act is a good explanation to the extent it provides information that, under the right circumstances, enables the audience to competently draw inferences that are needed to achieve the contextually salient purposes. Based on this, we propose the following schematic definition of explainability:

*Explainability:* in the conversational context *C*, a given phenomenon (model, system, prediction, …), *P*, is explainable by an explainer, *S*, to an audience, *A*, to the extent *S* is able to convey information to *A* that enables *A* to draw inferences about *P* that are needed to achieve the purposes that are salient in *C*.

The explainer can either be a human, possibly supported by some technical XAI tool for extracting relevant information, or a fully automated explanation-generator.^5^ The key point here is that explainability by this definition is always relative to a specific audience and contextual purpose. Without first specifying the relevant audience and purpose, there is no well-defined sense in which a given system is more or less explainable.

This definition forms the core of our framework. Its main function is to help elucidate what is at stake in different claims about explainability (or the lack thereof), by suggesting a series of heuristic questions. Given some claim about whether a system (or model, decision) is explainable, ask first: *to whom* does it need to be explainable and *why*, i.e., who is the *audience* and *what purposes* motivate this need? Second, what *inferences* does this audience need to be able to draw in order to achieve this purpose? Third, *what information* does the audience need in order to competently draw those inferences? Finally, *who* is supposed to supply this information, i.e., who is the *explainer*? The answers to these questions will determine to what extent the system is explainable, namely to the extent that the explainer is able to convey the necessary information to the audience. This heuristic, together with our definition of explainability, constitutes the framework we call *Explanatory Pragmatism*. In the rest of this section, we clarify a few of its key features.

As with the Mo/Jill fridge example in “Inferentialist view of understanding”, the context-sensitive elements of this definition, i.e. what the salient purpose, explainer and audience are, is determined by the *speaker context*, i.e. the conversational context within which an explainability claim is put forward and evaluated. The intentions, wants and needs of the explainer and audience themselves have no direct relevance, except insofar as they happen to be the ones discussing the explanation claim. It is because different speakers may have different audiences and purposes in mind that they risk talking past each other. By providing a framework for making these presuppositions explicit, Explanatory Pragmatism can facilitate cross-disciplinary communication and help resolve disagreements that arise from such misunderstandings.

This is not to say that the framework will automatically resolve all disagreements. In particular, there may remain substantive normative disagreements over which purposes it is important that the audience is able to achieve. Our framework is neutral with regards to those questions. Similarly, a given type of explainability may sometimes trade off against other important purposes. Again, Explanatory Pragmatism does not provide guidance as to how such trade-offs should be resolved. Disagreements will instead have to be resolved through independent normative arguments and value judgements. The value of our framework is that it forces us (designers, evaluators, etc.) to make these disagreements explicit such that they can be directly debated and potentially resolved.

As mentioned, we are not the first to highlight the importance of audience and purposes for XAI. Besold & Uckelman (2018) argue that a criterion for explainability is that the system can satisfy the user’s “subjective epistemic longing”, i.e., that it helps the audience learn things they desire to know. Tomsett et al. (2018) distinguish between different roles agents can play in the “machine learning ecosystem”, such as creators, examiners, operators or decision-subjects. They argue that the goals of a given agent depend on the role(s) they play, and that this in turn affects what kinds of explainability the agent requires to achieve those goals. Building on Tomsett et al., Zednik (2019) distinguishes different types of explanation-seeking questions that different stakeholders require answers to. For instance, he argues that system operators need to know *what* the system is doing, i.e., how it maps inputs to outputs, while decisions subjects (the individuals about whom predictions and decisions are being made) require answers to *why* its outputs are appropriate, i.e., what correlations in the environment make the outputs a reliable guide to a given decision.

Like Explanatory Pragmatism, these accounts define explainability to depend on audience and purposes. However, their starting point is the explanatory interests (desires, goals) *of the audience* (or generic roles, e.g., decision-subjects in general). By contrast, our framework starts from the purposes that are salient *in the speaker context* and asks what inferences different agents need to be able to draw to achieve those purposes. Thus, it highlights that debates about explainability can involve normative disagreements about what the audience *should* be able to do, rather than what the audience happens to want or desire.

Notice, finally, that our framework only gives a criterion for evaluating whether a system is explainable to a given audience, and a heuristic for identifying what kinds of information the audience needs for the system to be explainable. It does not entail the further claim that conveying this information would count as an explanation *of* the system or, more generally, what type of explanation-seeking question it would be an answer to.^6^ Similarly, we do not assume that the solution to lacking explainability is always to provide more information. Sometimes, a better solution is to change the system (e.g., making it simpler and easier to understand) or improve the audience’s inferential abilities by some other means. (We discuss an example of the latter in “Clinical reasoning”). In other words, if we take explainability to mean something like “the things that need to be explained, can be explained”, there are two ways to ensure this: either make sure more things can be explained (i.e., provide more information) or make sure that fewer things need to be explained (i.e., change the system or the audience’s inferential abilities).

### Potential objections

We flesh out Explanatory Pragmatism further in the following sections, by using it to introduce and distinguish several different kinds of challenges to explainability (“Challenges to explainability”) and by applying it to a concrete case study (“Purposes and audiences in medical AI”). First, however, let us address a few potential objections. For, on this type of contextualist, pragmatist view, is there any objective basis or guidelines for deciding how good a given explanation is? Worse yet, since we impose no general restrictions on the salient purposes, does our view entail that anything can count as a good explanation? If so, doesn’t that trivialise the notion of explanation beyond the point of usefulness?^7^

Regarding the first point, our framework does have an objective basis for evaluating explanations, namely whether they improve the inferential abilities that the audience needs to achieve the salient purpose. Now, as mentioned, there is no determinate answer to how good a given explanation is independently of such purpose and audience. But once these are specified, Explanatory Pragmatism provides clear, objective guidelines for determining the quality of an explanation.

On whether this means that *anything* can count as a good explanation, the answer is no: only information which improves the audience’s inferential abilities can be explanatory. Nonetheless, in many cases there will still be some ingenious way to specify a purpose and an audience, such that a given type of information will improve the inferential abilities the audience needs to achieve the purpose. To take a vivid example (adapted from Kitcher and Salmon, 1987), suppose an AI system predicts that a patient will not recover from a coma, and their relatives ask why. Suppose, moreover, that the only contextually salient purpose is to enable the relatives to articulate a coherent narrative about their loved one’s illness and trajectory, irrespective of the truth or predictive accuracy of this narrative. If the relatives believe in astrology, information about the current position of the stars and their (supposed) influence on the patient’s specific condition might serve this purpose well. Then, according to our framework, this type of astrological information would count as a good explanation.

While true, this implication is less radical than it may sound. First, the fact that it counts as a good explanation *in that context* does not entail that it counts as good in all other contexts. Second, even when the ability to articulate a coherent narrative is a salient purpose, it is rarely the only one. Presumably, we are not just looking to help the relatives articulate a coherent narrative, but also one that is (at least to some extent) true or accurate. Assuming the position of the stars does not, in fact, influence or predict terrestrial events in anything like the way astrology postulates, and the model does not rely on this type of information either, no amount of astrological explanation would serve those purposes. Thus, in most contexts conveying astrological information would *not* be a good explanation.

An analogy with other context-sensitive terms may be helpful here: whether something counts as ‘big’ depends on a contextually salient standard. 180 centimetres is tall for a miner, but short for a basketball player. 18 centimetres is big for an insect, but not at all for humans. 260 picometers is big for an atom, but small for most other things. However, this does not mean that there is no objective basis for calling things big or that there are no guidelines for determining whether a given entity is big, once the relevant standard is clear. Likewise, it is true that almost any size can count as big in some context, but this does not mean that they count as big in all other contexts. In most contexts, 260 picometres is very small indeed.

Granted, our framework is more permissive than what more restrictively minded philosophers might accept. But is it therefore useless? That depends (of course) on what we want to use the notion of explanation for. The restrictively minded philosopher, we suspect, wants to use it as a tool for criticism, by denying that astrology provides good explanations. If such explanations can count as good in some contexts (however unusual), that might seem to blunt the critical force of explanation claims. However, Explanatory Pragmatism leaves plenty of space for criticism.

First, if astrological explanations are evaluated in a context where some degree of truth or predictive accuracy is a salient goal—which is arguably the case in most scientific and medical conversations—one can point out that the proposed explanations lack those qualities. A proponent of astrology can of course retort that they believe their theory to be true (or accurate). But the same disagreement would arise even if we built some truth/accuracy constraint into the definition of ‘good explanation’.

Second, if a proponent of astrology only claims that their explanations are good relative to the purpose of articulating internally coherent narratives, one can highlight that there are many other important purposes that astrological explanations do not serve well. Furthermore, one can plausibly argue that *only* giving patients and their relatives the ability to articulate coherent narratives is paternalistic and potentially manipulative. It is a widely accepted principle of medical ethics that patients should be given truthful information about their condition and the basis upon which medical decisions affecting them are made. Here, the underlying disagreement is revealed to be a normative, ethical one, namely about what purposes we should prioritise and thus what kinds of understanding our explanations should aim to provide. If anything, this strikes us as a stronger basis for criticism than whether astrological information can ever count as explanatory.

Finally, we regard it as a virtue of Explanatory Pragmatism that it leaves space for these kinds of normative disagreements to be debated directly and explicitly, rather than packing them into a dispute about what counts as good explanations. For instance, it could be argued that the above-mentioned principle can be defeated or outweighed in certain cases. In the case of terminally ill patients, for example, it might be argued that the overriding priority should be to help them articulate a coherent and meaningful narrative about the end of their life. Regardless of whether one accepts this argument, we want our framework to leave space for the view that, in some cases, it would be inappropriate to insist on truthfulness/accuracy as a criterion for good explanations.

To summarise, if any critical potential is sacrificed by Explanatory Pragmatism, it is negligible and more than made up for by its advantages, namely: (1) that it accommodates the plurality and context-sensitivity of explanations within a unified framework, and (2) that it requires underlying factual and normative disagreements to be made explicit, thereby facilitating communication and preventing misunderstandings between different stakeholders and disciplinary communities.

## Challenges to explainability

In our framework, the guiding question in determining whether an AI system is explainable is whether the audience has information that enables them to draw contextually relevant inferences. Building on earlier literature, we distinguish several different ways this can fail to be the case.

Some challenges arise due to features of the agents involved, i.e., the explainer and audience. Consider for instance:

*Secrecy*: even if the relevant information is available, the explainer may not be willing, permitted or designed to convey it to the audience. This can be for legitimate purposes, such as preserving trade secrets or other confidential information (Burrell, 2016).

*Technical literacy*: even if the explainer conveys the information, the audience may not be familiar enough with the relevant vocabulary to fully comprehend this information. For instance, they may not be familiar with certain types of mathematical formalism or the definition of technical terms, such as what it means for a model to be ‘optimised’ for a certain goal (Burrell, 2016).

While these are both important, our focus in this paper will be on challenges that arise even if there are no restrictions on the types of information that can be conveyed by the explainer or comprehended by the audience.

A further type of challenge arises due to intrinsic features of the model, in particular its size or complexity:

*Complexity*: even if the audience can comprehend the information, it may be too complex for the audience to effectively and efficiently reason with or about. For instance, advanced machine learning models, such as a deep neural networks or large ensemble models, can take hundreds or thousands of input variables and use these to calculate a highly nonlinear and non-monotonic function (Selbst & Barocas, 2018, 1094-96), meaning that there are no simple, overall rules for whether increasing a given input variable will increase or decrease (and by how much) the probability of a given decision.

When a system becomes too large or complex, it can become infeasible for humans to competently draw inferences about its behaviour (at least within timescales that do not defeat the point of automating decision making in the first place). For instance, it may become infeasible to meaningfully follow and trace how the inputs get transformed into a given output, or to make even qualitative predictions about the behaviour of the system given different inputs.

Finally, often the inferences needed to achieve certain goals require relating the model to other relevant information.^8^ We can distinguish two potential problems here.

*Semantic mapping:* even if the audience is able to effectively and efficiently reason about what goes on within the model itself (e.g., how a given decision depends on the input), they may not be able to meaningfully relate this information to any of their other representations. Thus, they are unable to compare and integrate this information with any of their pre-existing knowledge.

*Domain knowledge:* even if the audience is able to relate information about the model to other representations of the world, these representations may not be sufficiently connected to other relevant pieces of background knowledge to allow the audience to competently make the inferences they need to achieve the contextual purpose.

Another way to spell out the difference between the two challenges is this. Semantic mapping concerns the *links between the model and other representations of the world*: the extent to which the audience can translate or interpret information about the model in terms of their other knowledge of the world. By contrast, domain knowledge concerns the *links between these other representations*: the extent to which the audience is able to make further, contextually relevant inferences once they have interpreted the model (or its predictions) in terms of a given set of representations.

Here is a toy example to illustrate this distinction. Suppose we have trained a machine learning system to predict where and when traffic congestion is likely to arise, based on input data from CCTV images of current traffic. We are able to extract the decision rules of the system and discover that predictions of congestion at junction J58 rely on a simple linear rule such that activity in a certain collection of pixels, *F*, makes future congestion at J58 more likely. Consider now three different scenarios:

1. * F* consists of a diffuse collection of pixels from several different cameras.
2. * F* consists of a region of a specific camera, depicting the turn-off onto road R15, one of the main roads leading to J58.
3. *F* consists of a region of a specific camera, depicting a car park in a different part of the city than J58.

In the first scenario, *F* is not related in any meaningful way to the concepts we use to understand traffic flows. Despite the simplicity of this decision rule, it does not help us understand the system’s ability to predict congestion at J58. It lacks an adequate semantic mapping.

By contrast, in scenario 2 we are able to relate the decision procedure of the system to our other representations of the traffic system, putting us in a better position to understand the algorithm’s predictive power. We can for example infer that it predicts congestion at J58 by monitoring the amount of traffic going onto one of the main roads leading to J58. This in turn allows us to make predictions about the performance of the system under different circumstances, e.g. whether it will continue to be reliable if roadworks cause traffic on R15 to be diverted around J58.

However, having a meaningful representation is not sufficient to make these inferences: we also need the right kinds of domain knowledge about how traffic flows work. This is illustrated by scenario 3. Here, *F* does map onto our concepts and representations of the traffic system, so the decision rule has a good semantic mapping. But we lack the domain knowledge to infer what kind of causal chain might make activity in the parking lot predictively relevant to future congestion at J58.

To summarise, we have distinguished five potential challenges to explainability: (i) secrecy, (ii) technical illiteracy, (iii) complexity, (iv) inadequate semantic mapping, and (v) lack of relevant domain knowledge.^9^ What they have in common is that they in some way or another limit what inferences can be drawn in relation to the system or its decisions.

Improving the explainability of a system can involve overcoming any of these challenges. For instance, if technical literacy poses a challenge, improving explainability might involve high-level explanations of the nature and limitations of machine learning, while, if complexity is the issue, it may be possible to provide some kind of simplified (e.g., partial, localised or approximate) representation of the model or the relation between its inputs and outputs.^10^ Similarly, if semantic mapping or domain knowledge is the issue, what is needed may be explanations of how the model relates to model-external features of the world. Furthermore, as highlighted in “Explanatory Pragmatism﻿”, sometimes the best way to improve explainability will involve changes to the model or improving the audience’s inferential abilities, rather than providing explanations. Overall, whether any of these potential challenges in fact undermine the explainability of a system, and how best to overcome them, depends on the salient purposes, the audience’s background knowledge and other factors shaping their inferential abilities.

## Purposes and audiences in medical AI

In this section we illustrate how our framework can be applied within the medical context, by using it to evaluate the explainability of the consciousness recovery prediction model introduced in the “Introduction”. We start by reviewing why the authors regard this model as explainable. We then consider three salient purposes for making this model explainable: (i) further research, (ii) deployment decisions and (iii) clinical reasoning. While we do not provide an exhaustive analysis of what explainability would amount to in each case (that would go far beyond the scope of a single paper), we aim to illustrate the flexibility of our framework by identifying the varying requirements for explainability that arise across these contexts. Finally, we briefly summarise and discuss some further lessons from these analyses (“Summary”).

### Explainability in Song et al.

Song et al. highlight three factors in support of their claim that their model is “interpretable”. First, their model is fairly simple: in fact, it consists of a linear function of just nine input features. Moreover, they were able to use a technique called Significant Multivariate Correlation (sMC) to estimate the relative importance of each input feature for the model’s predictions. Second, the input features of the model represent either clinical characteristics (aetiology, patient age and duration of condition) or features of the patient’s brain activity extracted from an fMRI scan of the patient. The latter represent either activity in specific well-defined brain areas or the functional connectivity between these brain areas. These include, for instance, areas in the default motor network (DMN), the executive control network (ECN) and the functional connectivity between parts of these two networks. Third, the authors highlight in their Discussion section that some of these brain areas have been related to disorders of consciousness in previous studies. In particular, they highlight that correct communication between DMN and ECN is “thought to be very important for optimal information integration and cognitive functioning” (p. 20) and that “A recent study reported that negative functional connectivities between the default mode network and the task-positive network were only observed in patients who recovered consciousness and in healthy controls, whereas positive values were obtained in patients with impaired consciousness” (p. 20).

In terms of the typology developed in “Challenges to explainability”, what the authors are pointing out is that their model has

- Low complexity: the model is simple, and it is easy to determine the contributions each feature makes to model predictions;
- a good semantic mapping: the input features represent the world in the same way that a human neuroscientist would;
- at least some domain knowledge connections: the authors were able to reason about the relation between their results and previous studies of the neural mechanisms involved in DoC.

Is this enough to make the model (or its decisions) explainable? As we have argued, this question cannot be fruitfully addressed in isolation from any particular contextual purpose that a given audience needs to achieve.

Song et al. mention two audiences that could benefit from the explainability of their model: scientists and physicians (p. 21). They also suggest a purpose, namely to “reassure [them] about the significance of different predictors” (ibid.). However, there are arguably many different reasons these audiences might need such reassurance. For the purposes of our analysis, we distinguish three salient (but non-exhaustive) purposes.^11^ First, we assume that by ‘scientists’, the authors mainly have in mind medical researchers and neuroscientists who need to use or build on the results of this study for further research on DoC. Second, for physicians, we consider two potential purposes: deployment decisions and clinical reasoning.

### Further research

For the purpose of conducting further research, the relevant inferential abilities that researchers need include being able to see how the results of this study relate to other literature on the neural mechanisms involved in DoC. For instance, scientists need to be able to reason about the theoretical implications of the correlations found by Song et al., and to evaluate whether these are consistent with previous research. The model seems well-suited for this: first, the low complexity makes it easy to identify and reason about the implications of the correlations built into the model (or at least, easier than if it had been a high-dimensional model with many nonlinear interactions between the input features); second, the semantic mapping makes it easy to compare the model to other relevant studies, as the authors themselves do.

Thus, for the purpose of conducting further research by an audience of medical researchers and neuroscientists, the model can plausibly be said to have a high degree of explainability.

Interestingly, in the research context, more extensive semantic mappings (or more extensive domain knowledge) might detract from other important goals. Though a key to effective explanations of machine learning systems is integration of their outputs with existing knowledge (Gil, 2021), in many cases, the aim of using machine learning in research is to discover *new* correlations that extend or contradict our existing beliefs. Thus, there is a potential trade-off here. On the one hand, if we were to always insist on complete semantic mappings so that all aspects of the model could be tied neatly to our existing understanding, we would miss out on new discoveries. On the other hand, if a new correlation cannot be related in any way to existing knowledge, how would we be able to recognise or make sense of it?

Exactly how to balance this trade-off will involve contextual value judgements. In more exploratory research, a quite minimal semantic mapping may suffice, as developing more detailed understandings can be left for future research. In deployed contexts, where the health of patients is on the line and novel discoveries are at most a secondary concern, it is probably best to skew conservatively towards integration with accepted knowledge and clinical practice. However, as we emphasised in “A framework for explainable AI”, the Explanatory Pragmatism framework is not designed to directly adjudicate such decisions, but rather to help make explicit the value judgements involved.

### Deployment decisions

In some contexts, physicians will have to make decisions about whether to deploy the model in a new hospital, either individually or in the role of a hospital administrator or health policy advisor (such as Chief Medical Officers in the UK). To constrain the case, let us assume that the population at the new hospital differs in a number of potentially relevant factors (in, for example, genetic characteristics, socio-economic status, age distribution, environmental exposures, etc.) from the population the model was tested on. We will also assume that the decision specifically concerns whether to deploy the model now, in its current form, or to wait for the model to be tested and possibly retrained on data from the new application context. The latter option would, of course, reduce the risk but gathering new data would also be costly and time-consuming, thus delaying any potential benefits that could be gained from the system.

For this purpose, the inferential capacities needed include being able to determine how likely the model is to perform reliably in this new setting and to evaluate the overall risks and benefits deploying the model would entail. This is necessary to decide, e.g., whether decisions to remove life sustaining interventions can responsibly be based on, or at least informed by, the model’s predictions. As we have stipulated, the physician needs to be able to determine this from an explanation of the current model rather than by testing or retraining the model on new data. However, the model did achieve a high predictive performance at the hospitals in both Beijing and Guangzhou. The question, then, is whether the physician is warranted in expecting a similarly high performance in the new population. In other words, does she have good reason to believe that the correlations that the model relies on, e.g. between activation in the default motor network and consciousness recovery, also obtain at the new hospital?

Inferences about whether the results from a given study apply in a new context are called *extrapolation* in philosophy of science, where their logic has been extensively studied.^12^ A general lesson from this literature is that extrapolation always relies on some background knowledge or theory, in addition to information about the results in the study population. In simple cases, we might know that two populations are generally similar (e.g. if one is a representative sample of the other). However, in cases like the one considered here, where the populations are known to be dissimilar, more detailed information about the conditions underwriting the performance achieved in the study population is required. For instance, there may be features of the study population that were necessary for producing a given correlation that are missing in the target population. Similarly, there may be additional features of the target population that modulate or block the same correlations from obtaining. Without a good understanding of which features could influence the correlations that the model relies on, it is difficult to make reliable inferences about whether any of the known differences between the two populations are likely to affect the model’s performance and, similarly, if there are any unknown differences that might be relevant.

Returning to the consciousness recovery model, to be warranted in applying the model at the new hospital a physician needs some information about the world, namely what neural mechanisms and processes that underpin the correlations embedded in the model. Despite the simplicity of these correlations, they may nonetheless be the result of highly complex neural processes involving many features not represented in the model itself. For instance, as noted above, one of the most important predictors in the model is activity in certain areas within the DMN and their functional connectivity to areas in the ECN. However, the interpretation of DMN activity remains controversial within neuroscience (e.g. Harrison et al., 2008). A number of factors, both physiological and environmental, have been shown to affect baseline DMN activity. These include childhood poverty (Sripada et al., 2014), being an experienced meditator (Brewer et al., 2011), off-task thought and mind wandering (Zhang et al., 2019), depression (Wise et al., 2017), antidepressants (Posner et al., 2013), systemic inflammation (Marsland et al., 2017), Alzheimer’s and cognitive decline (Zhang et al., 2020). It is possible that these factors could change DMN activity post-DoC as well. Thus, if the distribution of these factors—or other currently unknown factors affecting baseline DMN—differs significantly between the study and target population, this could invalidate the model’s predictions. Even if overall predictive accuracy remains similar, there may be sub-populations within the target population for whom the model’s performance declines significantly.

The point is, given the current state of neuroscientific knowledge we simply do not know whether this will be the case. Thus, due to this lack of domain knowledge, we cannot explain the reliability of the model sufficiently well to allow physicians making deployment decisions to fully determine whether Song et al.’s model is likely to perform well in new contexts. So, for the purpose of deployment decisions to new populations, the model is not fully explainable. This is not to say that it is completely *un*explainable. As the above discussion illustrates, the semantic mapping together with our existing domain knowledge does allow us (at least to some extent) to reason about what the potential risk factors are. Whether this is sufficient to warrant deploying the model in a given setting will depend on the other potential risks and benefits at stake in that context.

### Clinical reasoning

Where the model is deployed in clinical practice, physicians who will be relying on its predictions face a further challenge, namely how to integrate these predictions with other pieces of evidence into their overall clinical reasoning. For example, suppose that in addition to the predictions of Song et al.’s model, the clinician also orders a blood test for a certain enzyme which (let us assume) is known to correlate with consciousness recovery. If the model predicts a low score for consciousness recovery, but the blood test comes back positive for a given patient, is this sufficient to dismiss the model’s prediction? Similarly, if the blood test and the model’s predictions are both positive, should that make the clinician extra confident? If so, by how much? For instance, if the model predicts a high score of 18 for one patient who tests negative for the enzyme, while another patient receives a lower prediction of 13 but tests positive for the enzyme, who should we prioritise for therapeutic interventions?

For the purpose of clinical reasoning, then, a salient contextual purpose is to make inferences about how to integrate the predictions of the model with other pieces of evidence. We outline two challenges to achieving this.

The first concerns how probabilistically independent the two types of evidence are, i.e. whether knowing the result of one makes the other more likely to be observed. For instance, if the enzyme turns out to be produced by the types of brain activity that the model relies on, observing both together would be less surprising and therefore add less confirmation. By contrast, if the enzyme is produced by a completely separate physiological process, consilience between the two pieces of evidence might significantly increase our confidence in the prediction. Now, for any two types of evidence it may of course be possible to do additional testing to estimate how much they correlate. However, if this has to be done for many different types of evidence, the combinatorial explosion would quickly make this strategy infeasible. Additional knowledge about the biological mechanisms underlying these correlations—as in the example above—can help overcome this challenge, allowing the physicians to reason about which pieces of evidence are more likely to be dependent. Here it is a lack of domain knowledge that limits explainability.

The second challenge is that these types of formal knowledge may not be sufficient to figure out how to weigh the information provided by these machine learning models, even once validated. Some clinicians (e.g. see Norman, 2006; Chin-Yee & Upshur, 2018) maintain that experiential knowledge has been underemphasized in the teaching of clinical reasoning. Clinical practice is not just a matter of explicitly reasoning through the evidence, but also relies on recognition and clinical judgement. The latter is based on tacit knowledge which can arguably only be developed through practical experience. Thus, in addition to formal explanations, practical work is likely needed to help clinicians develop sufficient experience of how to integrate machine learning models into clinical practice, including how to weigh their predictions against other types of evidence and judgment that are available to them. As this experience develops, clinicians may in turn discover further knowledge gaps that need to be filled in order to improve their understanding of machine learning models. Thus, there will likely be an iterative interplay between formal explanation and clinical experience, through which new requirements for explanation will need to be developed and discovered.

An illustration of what this might mean in practice is given by the case study by Sendak et al. (2020) discussed in “Current directions in Explainable AI”. In this case study, a specialised team of two nurses was trained to continuously monitor their deployed machine learning model and translate its outputs into actions for the different medical specialists treating the patient. This translational work is non-trivial. These nurses’ specialisation consisted not just in their formal knowledge of the model, but also their clinical experience of working with the model in practice. This was critical to ensure that the requirements for explanation are fully discovered and realised.

So, for the purpose of clinical reasoning, the model by Song et al. is not fully explainable. In order to make it more explainable, additional domain knowledge is needed in order to weigh the predictions against other clinical evidence and further practical and experiential knowledge of working with the model.

## Summary

The preceding analyses illustrate some (though not all) of the ways context and audience matters to whether an AI system counts as explainable. For the purpose of further research, we saw that the low complexity of the consciousness recovery model and its good semantic mapping made it sufficiently explainable. The limited available domain knowledge did not pose a challenge to explainability in this context. This turned out not to be the case for the other two purposes. In the case of deployment decisions, what was lacking was knowledge of the support factors that underpin the reliability of the model’s prediction and how these support factors relate to the potential deployment population. In the case of clinical reasoning, there was insufficient knowledge about the relationship between the biological mechanisms that this model relies on and those that are measured by other kinds of evidence. Thus, although the lack of explainability in both cases stemmed from limited domain knowledge, different kinds of domain knowledge was lacking in each case. We also highlighted a further challenge in the case of clinical reasoning, namely that clinicians often also need certain kinds of practical and experiential knowledge to integrate new forms of evidence into their decision-making processes.

## Conclusion

In this paper, we have proposed a pragmatist account of AI explainability. We have used it to classify five distinct challenges to explainability, as well as to elucidate the requirements for adequate explanations that arise in medical contexts with regards to three different purposes.

A key takeaway from our analysis is that the problem(s) of explainability cannot be exhaustively solved in the abstract. There is not going to be a single approach to XAI that can simply be applied off-the-shelf to generate adequate explanations for any given AI system. Close attention to the context of application is necessary. In particular, we have highlighted three types of contextual detail that need to be considered. First, explainability on our account is relative to a specific audience and purpose. A strength of our framework is that it makes visible disagreement about which purposes are important and provides a way of analysing what is needed from explanations for each. Second, challenges to explainability often stem from the state of our domain knowledge, rather than (merely) the intrinsic complexity of the model or the limitations of the explainer or audience. Finally, as we discussed in relation to clinical reasoning, certain kinds of experiential knowledge are often necessary for a given audience to obtain the necessary inferential abilities, in addition to formal explanations.

Thus, a context-sensitive and iterative approach to the discovery and development of explainability requirements will often be needed. As we have argued, the framework defended in this paper is both unified and flexible enough to guide such explorations.

## Data Availability

Not applicable.

## Footnotes

1 DoC is defined as conditions where a patient’s capacity for arousal or awareness is absent or inhibited. These mainly include coma, persistent vegetative state and minimally conscious state.

2 Specifically, a regression model was first trained to predict patients’ score on the Coma Recovery Scale-Revised (CSR-R), which combines six sub-scales estimating auditory, visual, motor, oromotor, communication, and arousal functions. To produce a binary classification, a cut-off point on the predicted CSR-R scores was then calculated, above which patients were predicted to recover consciousness (defined as a score of 3 or higher on the Glasgow Outcome Scale). The cut-off point was selected to maximise the sum of true positive and true negative classifications in the training data.

3 There is considerable terminological variation in these literatures. Some use terms such as ‘interpretability’ and ‘explainability’ more or less interchangeably, while others define them to mean different things (e.g. Lipton 2017). Our account is framed in terms of ‘explainability’ (for reasons that will become apparent below) and we will mainly be using this term, except when quoting others.

4 There is a debate over whether abilities are an essential part of understanding. Some argue that inferential and practical abilities are simply the typical consequence of the right kinds of knowledge, e.g. of inferential or explanatory relations (Kelp, 2015; Sullivan, 2018). Others argue that these types of knowledge are simply the typical means of achieving the right kind of abilities (e.g. Stuart, 2018; Wilkenfeld, 2013). For our purposes, it is not crucial how this debate is resolved. In practice, robust inferential and practical abilities can usually only be achieved through having at least some relevant knowledge; conversely, we can usually only know that someone has certain beliefs or representations if they manifest some relevant abilities.

5 We are open to the explainer and audience in some cases being the same individual. We would construe this as a limiting case of our definition where “*S* is able to convey information to *A*” reduces to “*A* is able to obtain information”.

6 Many thanks to an anonymous referee for alerting us to this ambiguity in our view.

7 We are grateful to an anonymous referee for raising these points. The same reviewer also suggested we discuss a variant of Kitcher and Salmon﻿’s (1987) astrology example. Notice, since most of Kitcher and Salmon’s arguments focus on the details of Van Fraassen’s (1980) view, they do not apply to our account.

8 Other commentators have emphasised that explainability (or the lack thereof) is often a product of the model’s relation to other background information or representations of the world, including Felten (2017), Selbst & Barocas (2018), Sullivan (2019), Zednik (2019), Heinrichs & Eickhoff (2020).

9 A further challenge arises for systems that continue to change whilst being deployed, e.g., due to online-learning or frequent manual updates. Here, even if an explanation can be given at one time, the system may change in ways that render the explanation it provides no longer adequate. We will not discuss this challenge further.

10 See Erasmus et al. (2020) for a useful typology of this type of explanation.

11 We say non-exhaustive, as there are certainly other audiences and purposes for which explanations can be required. For reasons of space we have chosen to focus on a few in order to illustrate the utility of our framework. However, it is worth briefly mentioning two further important purposes that have been highlighted as motivations for requiring explainability in medical contexts. First, enabling patient-centred care and patient autonomy through informed consent and shared decision-making practices arguably requires its own type of explainability (Bjerring & Busch, 2020; Keeling and Nyrup, manuscript). Second, the issue of algorithmic bias remains an important problem (Buolamwini & Gebru, 2018; Crawford, 2017). For example, in medical contexts, algorithms have been found to systematically underestimate the healthcare needs of Black patients relative to white patients (Benjamin, 2019; Obermeyer et al., 2019). Explanations that describe the construction and workings of AI systems are arguably an important tool for the purpose of auditing and ensuring fair systems, for which the relevant audiences include designers, regulators and potentially the wider public. Exploring the implications of our framework for these is an important task for future research.

12 See e.g. Steel (2007), Cartwright (2011, 2013) and Khosrowi (2019) for general discussion, and Pietsch (2015, 2016) and Northcott (2020) for analyses focused on machine learning and data-driven modelling. The extent to which a result can be extrapolated to new contexts is sometimes called its ‘external validity’.
